# Texture improvement and *in vitro* digestion modulation of plant-based fish cake analogue by incorporating hydrocolloid blends

**DOI:** 10.1016/j.crfs.2024.100775

**Published:** 2024-05-23

**Authors:** Felicia Zhi Wen Peh, Lin Zhao, Yin Yin Chia, Cheryl Kwoek Zhen Ng, Juan Du

**Affiliations:** a Food, Chemical and Biotechnology Cluster, Singapore Institute of Technology, 10 Dover Drive, Singapore, 138683, Singapore; b Department of Food Science, Purdue University, 745 Agriculture Mall Dr, West Lafayette, IN, 47907, USA; c Sengkang General Hospital, Singapore Health Services, 10 Hospital Boulevard, Singapore, 168582, Singapore

**Keywords:** Plant-based seafood analogue, Food hydrocolloids, Brown rice protein isolate, Pea protein isolate, Amino acid release, Rheological properties

## Abstract

Hydrocolloids have proven effective in improving the texture of surimi gels, yet their application in plant-based seafood analogues remains underexplored. This study aimed to develop a hydrocolloid blend comprising methylcellulose (MC), curdlan gum (CG), and high-acyl gellan gum (GG) to achieve a surimi-like texture in plant-based fish cakes (PBFC) made from brown rice and pea protein isolates. The research showcased that higher MC concentration boosted protein powder's heated oil holding capacity, while CG concentration increments lowered it. However, heated water holding capacity remained stable despite changes in MC and GG levels. Incorporating hydrocolloids elevated PBFC moisture content, decreasing expressible moisture and oil amounts with rising MC, CG and GG concentrations. PBFC hardness increased with higher hydrocolloid levels and was influenced by temperature, while springiness remained unaffected. GG helped maintain storage modulus (G′) during PBFC cooling at higher concentrations, whereas the opposite effect was observed for MC. Analytically, higher MC concentrations reduced protein digestibility, while increased GG concentrations appeared to enhance it. Microstructural analysis corroborated these findings, with more protein aggregates in PBFC containing 3.8% MC and fewer in PBFCs with 6% CG and 3% GG. Consumer evaluations indicated that PBFC formulated with 1% MC, 3% CG, and 1.5% GG matched the springiness of commercial surimi-tofu fish cake, though it received slightly lower overall liking scores. In conclusion, the combined use of these three hydrocolloids demonstrated the potential to enhance the physical properties of PBFC and modify protein digestibility, offering insights into the development of innovative plant-based seafood analogues.

## Highlights

• The combined use of gums enhanced plant-based fish cake's physical properties.
• Higher content of gums reduced expressible moisture and oil amounts.
• Gellan gum helped maintain storage modulus during cooling at higher concentration.
• Increased methylcellulose concentration decreased protein digestibility.
• Higher concentration of methylcellulose resulted in denser protein aggregates.

## Graphical abstract

**Figure:**
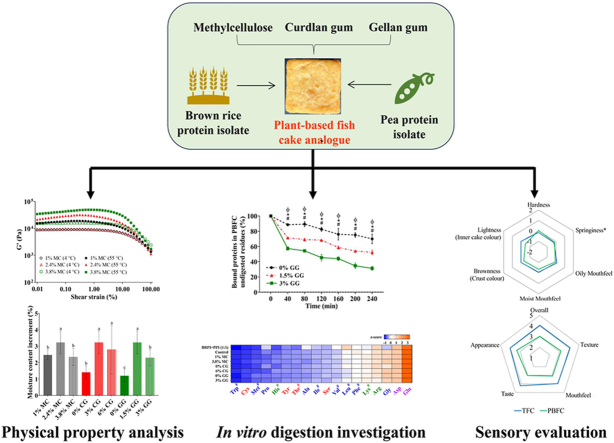

## 1 Introduction

Surimi, derived from mechanically processed fish flesh, is composed of stable myofibrillar protein. It serves as the primary ingredient for seafood products, like fish balls, fish sausages, and fish cakes. The surimi-based seafood products are widely favoured for their low cholesterol, abundant protein, and distinctive texture (Li et al., 2022). Although they are well-received, the growing popularity of plant-based seafood alternatives, driven by concerns about environmental sustainability, overfishing, and ethical considerations, is creating fresh prospects within the food industry (Yadav et al., 2021).

When seeking a plant-based protein source, brown rice protein isolate (BRPI) stands out as a promising option due to its hypoallergenic nature and impressive protein content (Zhao et al., 2023). However, its limited use in plant-based products can be attributed to its suboptimal surface-active properties and solubility (Phongthai et al., 2017). In this study, to enhance the qualities of BRPI, pea protein isolate (PPI) was chosen due to its superior functionality and a well-matched amino acid profile (Pinckaers et al., 2021). Given that texture significantly influences consumer acceptance of such meat analogues apart from protein sources, the incorporation of specific hydrocolloids becomes necessary to enhance the gel strength and functional characteristics of plant-based seafood alternatives.

Among the widely utilised food hydrocolloids in current meat analogues, methylcellulose stands out due to its capacity to bind ingredients, minimise oil absorption, and contribute to a juicy texture in the mouth. Methylcellulose is derived from cellulose by replacing some glucose units with methyl groups, resulting in a neutral and linear structure composed of β-(1–4) linked glucose units (Brady et al., 2017). Its distinctive feature of thermal reversible gelation, which occurs upon cooling, also aids in reducing cooking-related losses. Kyriakopoulou, Keppler and van der Goot (2021) discovered that methylcellulose's gelling and thickening properties contributed to stabilising emulsions and reducing oil and water loss in plant-based meat substitutes. On the other hand, curdlan gum, derived from bacterial fermentation, is a homopolysaccharide commonly employed as a thickener, gelling agent, and fat substitute in meat products (Wei et al., 2018). It consists of individual glucose molecules linked together through β-(1,3)-glycosidic bonds and maintains a neutral charge (Zikmanis et al., 2021). A previous study found that when added to ribbonfish meat gel, curdlan gum greatly enhanced the gel characteristics and sensory properties (Wu et al., 2015). Another hydrocolloid that has been thoroughly researched and applied in the food industry is gellan gum, which comes in two variations: low acyl and high acyl (Danalache et al., 2015). Low acyl gellan gum produces gels that are firm, lacing elasticity, and tend to be brittle, whereas high acyl gellan gum is known for its ability to create consistent textures by producing elastic, soft, non-brittle and stable gels (Morris et al., 2012). The properties of latter gels are linked to the attributes we aim to replicate in our plant-based fish cake analogue.

So far, numerous studies have been undertaken to investigate protein-polysaccharide interactions in the development of innovative plant-based foods (Ishaq et al., 2022). Examples include research on an egg omelette substitute using mixtures of chickpea flour, soy protein isolate and κ-carrageenan (Lu et al., 2022), as well as a plant-based fish ball analogue created using blends of soy protein isolate and konjac glucomannan (Ran et al., 2022). These investigations have emphasised how protein-polysaccharide interactions can significantly alter the macroscopic characteristics of food systems, leading to the attainment of favourable physicochemical properties.

However, no prior research has been carried out on the application of methylcellulose (MC), curdlan gum (CG), and high-acyl gellan gum (GG) in combination with BRPI and PPI for the development of plant-based fish cake (PBFC) analogues. Therefore, the primary objective of this research was to find an optimal ratio of these hydrocolloids that could provide a textural solution for producing PBFC with textures akin to traditional surimi-based counterparts and with high levels of consumer acceptance. Various physical attributes were subject to analysis, including texture profile, moisture content, expressible moisture and oil content, as well as the rheological properties. Furthermore, the *in vitro* protein digestibility of PBFC samples featuring various levels of MC, CG and GG incorporation was assessed, shedding light on the availability of amino acids in our PBFC products. To complement these findings, we employed confocal laser scanning microscopy (CLSM) to inspect the microstructures of selected PBFC samples, visually substantiating the textural and digestibility attributes observed earlier. This research forms the foundational work for broadening the scope of plant-based seafood analogues by exploring the potential of BRPI, PPI and various hydrocolloids.

## 2 Materials and methods

### 2.1 Materials

BRPI (Myprotein™, THG Limited, Manchester, England), PPI (Nutralys® S85F, Roquette Pte. Ltd., Singapore), methylcellulose (Benecel™, Ashland Pte. Ltd., Singapore), curdlan gum (FT1001, Alpha Colloids Pte. Ltd., Singapore), and high acyl gellan gum (Kelcogel® MA-60, CP Kelco Pte. Ltd., Singapore) were purchased from the respective company. Microbial transglutaminase (mTG) (Activa® TG-SR-MH, Ajinomoto Pte. Ltd., Singapore), fish flavourings (Symrise Fish Flavour 332251, Symrise Asia Pacific Pte. Ltd., Singapore, and Takasago Fish Flavour S2106973, Takasago International Pte. Ltd., Singapore), and flavour maskers (Symlife® Masking Flavour 812904, Symrise Asia Pacific Pte. Ltd., Singapore) were generously supplied by the respective company. Other ingredients, like salt, sugar, monosodium glutamate, pepper, dextrose monohydrate, and canola oil were purchased from a local supermarket. The specific formulations of PBFC containing different hydrocolloid levels are outlined in Table 1. The decision regarding the current varying levels of each hydrocolloid was informed by the results of the overall appearance and texture assessments carried out during our preliminary trials. These trials were necessitated by the fact that concentrations exceeding the current lower and upper limits of each hydrocolloid would either prevent paste formation during fish cake preparation or yield an unacceptable firm paste that hampered ingredients’ mixing.

**Table 1:** Concentration of ingredients used in PBFC and sample groupings based on hydrocolloid types and concentrations.

Ingredients (%)	Treatment groups based on hydrocolloid types and concentrations								
	MC	MC^a^	MC	CG	CG^a^	CG	GG	GG^a^	GG
	1%	2.4%	3.8%	0%	3%	6%	0%	1.5%	3%
GG	1.50	1.50	1.50	1.50	1.50	1.50	0.00	1.50	3.00
MC	1.00	2.40	3.80	2.40	2.40	2.40	2.40	2.40	2.40
CG	3.00	3.00	3.00	0.00	3.00	6.00	3.00	3.00	3.00
BRPI	5.00	5.00	5.00	5.00	5.00	5.00	5.00	5.00	5.00
PPI	5.00	5.00	5.00	5.00	5.00	5.00	5.00	5.00	5.00
mTG	0.10	0.10	0.10	0.10	0.10	0.10	0.10	0.10	0.10
Seasoning and flavours	4.42	4.42	4.42	4.42	4.42	4.42	4.42	4.42	4.42
Canola oil	12.50	12.50	12.50	12.50	12.50	12.50	12.50	12.50	12.50
Water	67.48	66.08	64.68	69.08	66.08	63.08	67.58	66.08	64.58

a Control group, which contained 2.4% MC, 3% CG and 1.5% GG.

For *in vitro* protein digestion, enzymes and bile extract porcine (B8631) were purchased from Sigma-Aldrich Pte. Ltd. (Singapore). The enzymes consist of α-amylase from porcine pancreas (A3176, EC 3.2. 1.1, 12 U/mg), pepsin from porcine gastric mucosa (P7012, EC 3.4. 23.1, 3940 U/mg), and pancreatin from porcine pancreas (P7545, EC 232.468.9, a mixture of multiple digestive enzymes including trypsin, lipase, amylase, protease and ribonuclease; 96.7 U/mg based on trypsin activity).

### 2.2 Preparation and processing of PBFC

The flow diagram for making PBFC is depicted in Fig. S1. After processing, the fish cakes were allowed to cool at ambient temperature for 30 min before further analysis.

### 2.3 Determination of heated water holding capacity (HWHC) and heated oil holding capacity (HOHC) of protein-hydrocolloid powder blends

Modified from Cheng et al. (2024), in HWHC analysis, 0.1 g of powder blend containing BRPI, PPI, MC, CG and GG based on their ratios in each formulation listed in Table 1 were added into a pre-weighed centrifuge tube, followed by addition of 40 mL deionised water. For control group, the powder blend contains only BRPI and PPI without hydrocolloids. The samples were then vortexed for 30 s, heated at 95 °C for 30 min and followed by centrifugation at 8500×*g* at 25 °C for 15 min. Similar procedures were performed for HOHC analysis. However, 1 g of powder blend to 10 mL of canola oil was used instead. For all samples after centrifugation, the supernatant was decanted, and the tubes were left to drain for 2 min before the residues were weighted. Both HWHC and HOHC can be calculated based on equation (1). All measurements were carried out in triplicates.

(1) $$\text{HWHC}\;/\;\text{HOHC}\;\left(g/g\right)=\frac{\text{Weight}\;\text{of}\;\text{residue}\;\left(g\right)-\text{Weight}\;\text{of}\;\text{dry}\;\text{powder}\;\text{blend}\;\left(g\right)}{\text{Weight}\;\text{of}\;\text{dry}\;\text{powder}\;\text{blend}\;\left(g\right)}$$

### 2.4 Physical property characterisation of PBFC

#### 2.4.1 Texture profile analysis

Texture profile analysis (TPA) was conducted using a Texture Analyzer (TA.XT Plus, Stable Micro Systems, Surrey, UK) with a flat-ended 36 mm aluminium cylinder probe (P/36). The parameter settings for texture analysis were referenced from Binte Abdul Halim et al. (2023) and Cropotova et al. (2021). Before the start of the analysis, the PBFC was cut into 1 cm × 1 cm × 1.5 cm cubes after crust removal. Samples were compressed twice at a strain of 60% of the sample height, at a test speed of 1 mm/s with 5 s holding time (can recover after compressing). The measurements were performed at two temperatures: 55 °C to simulate consumer consumption temperature and 4 °C which is the purchasing and storage temperature for fish cakes. The samples were either taken from the portable fridge (set at 4 °C) or a water bath (set at 55 °C) immediately before measurement. Both the portable fridge and the water bath were positioned alongside our texture analyzer, eliminating the risk for sample transportation to change temperature. For samples in the fridge or the water bath, it was also ensured that they had equilibrated to 4 °C or 55 °C before measurements. Each measurement lasted less than a minute, and preliminary tests were conducted to ensure that the sample temperature remained within ±0.5 °C before and after the measurement. Similar measurements were conducted for the commercial surimi-tofu fish cake (TFC) for benchmarking purposes. Emphasis was placed on hardness and springiness as these two attributes are representative of fish cake texture. For each sample at each temperature, quadruplicate measurements were conducted with samples made from replicate batches using the process in Section 2.2.

#### 2.4.2 Moisture content determination

Moisture content of the PBFC after manufacturing was determined based on the AOAC (2000) standard with slight modification. It was quantified via weight loss from drying 0.5 g of sample at 105 °C for 24 h in the drying oven (UN 55, Memmert, Germany). Quadruplicate measurements were conducted with samples made from replicate batches using the process in Section 2.2. Equations (2) and (3) depict the formulas for the moisture content of fish cake and its moisture content increment after processing, respectively.

(2) $$\text{Moisture}\;\text{content}\;\text{of}\;\text{fish}\;\text{cake}\;\text{after}\;\text{processing}\;\left(\%\right)=\frac{\text{Difference}\;\text{in}\;\text{sample}\;\text{mass}\;\text{after}\;\text{drying}\;\left(g\right)}{\text{Initial}\;\text{sample}\;\text{mass}\;\text{before}\;\text{drying}\;\left(g\right)}\times 100\%$$

(3) Moisture content increment after processing (%) = Moisture content of fishcake after processing (%) - Initial moisture content in formulation (%)

#### 2.4.3 Expressible moisture and oil content

The expressible moisture was determined in accordance with Zhao et al. (2024) with modifications. The PBFC was cut into 1 cm × 1 cm × 1.5 cm cubes which were placed on a piece of 2 cm × 2 cm weighed paper towel with another piece of weighed paper towel of similar size placed on top of each sample. The samples were compressed under a weight of 570 g for 10 min. Thereafter, the weight gain of the paper towels was measured. The paper towels were dried in the drying oven (UN 55, Memmert, Germany) at 105 °C for 24 h, a method modified from the AOAC (2000) standard for moisture content determination. The weight loss from the dried paper towels were assessed to be the expressible moisture content.

The weight difference from the dried paper towel to the initial unused paper towel were calculated to be the expressible oil content. Equations for expressible moisture and oil content are shown in equations (4) and (5), respectively. Quadruplicate measurements were conducted with samples made from replicate batches using the process in Section 2.2.

(4) $$\text{Expressible}\;\text{moisture}\;\left(\%\right)=\frac{W_{1}\;\left(g\right)\;-\;W_{2}\;\left(g\right)}{\text{Initial}\;\text{moisture}\;\text{content}\;\text{of}\;\text{sample}\;\left(g\right)}\times 100\%$$

(5) $$\text{Expressible}\;\text{oil}\;\left(\%\right)=\frac{W_{2}\;\left(g\right)-W_{0}\;\left(g\right)}{\text{Initial}\;\text{oil}\;\text{content}\;\text{of}\;\text{sample}\;\left(g\right)}\times 100\%$$

where W_0_ represents the weight of the initial paper towel before compression, W_1_ stands for the weight of the wet paper towel after compression, and W_2_ indicates the weight of the dried paper towel following the drying process.

#### 2.4.4 Rheological properties

Amplitude sweep and frequency sweep were conducted on the inner section of the PBFC with a rheometer (MCR302e, Anton Paar GmbH, Graz, Austria). A cross-hatched parallel plate of 25 mm diameter was used for the measurements to prevent sample slippage. The sample was sliced to a diameter of 25 mm and a thickness of 3 mm after crust removal. An amplitude sweep was first conducted on the samples with a constant frequency of 1 Hz and an increasing strain from 0.01% to 100% with 10 points per decade. From this test, the limit of the linear viscoelastic (LVE) region was found. After the destroyed sample was replaced, a frequency sweep was carried out with a constant strain of 0.1% strain (within LVE region) and an increasing frequency from 0.1 Hz to 50 Hz with 10 points per decade. Samples were evaluated at 55 °C and 4 °C. For each sample, triplicates were measured for each analysis at each temperature.

### 2.5 In vitro protein digestion

#### 2.5.1 Protein digestion progress during gastric and intestinal phase

Following the INFOGEST 2.0 standardised *in vitro* digestion protocol, as described by Brodkorb et al. (2019), we pre-made simulated digestion fluids according to the prescribed electrolyte concentrations in the protocol. Enzymes for each digestion mixture were added on the day of the experiment. Electrolyte stock solutions and simulated digestion fluids for each digestion phase were prepared as outlined in Table S1 and Table S2.

The PBFC were cut into 2 mm × 1 mm × 1 mm cubes. Prior to the experiment, the samples and all simulated digestion fluids were pre-warmed to 37 °C in a water bath. The oral phase digestion commenced by adding 3 mL of well-mixed simulated salivary fluid (SSF) with α-amylase to 3 g of the sample. The mixture's pH was adjusted to 7.0, and it was then incubated in a shaking water bath (200 rpm, 37 °C) for 2 min before transitioning to the simulated gastric digestion phase. The products from the oral phase were supplemented with 6 mL of simulated gastric fluid (SGF) with pepsin, which was warmed to 37 °C in advance. The pH of the resulting mixture was then lowered to 3.0, and then the mixture underwent continuous digestion for 120 min in a water bath (200 rpm, 37 °C). To assess the progress of protein digestion at various time intervals during the gastric digestion phase, the corresponding gastric digested mixtures were extracted at 40, 80, and 120 min, respectively. Subsequently, 6 mL of Milli-Q® system-generated Ultrapure type I water at 100 °C was added to swiftly halt the digestion process. The samples were further exposed to a 10-min heat treatment in a 95 °C water bath to ensure full termination of digestion.

The intestinal phase commenced for the remaining samples after 120 min of gastric digestion by introducing 12 mL of simulated intestinal fluid (SIF) with pancreatin and elevating the pH of the mixtures to 7.0. Similarly, to assess protein digestion progress during the intestinal phase at various time markers, digested samples were retrieved at 160, 200, and 240 min respectively and terminated using the same method described earlier.

To assess the progression of protein digestion at various time points, we analysed the protein contents in the remaining undigested PBFC residues at each time point, employing methods adapted from Fasuan et al. (2018). The residues were separated from the digested mixtures following the termination of the digestive reaction and rinsed with Ultrapure type I water (1:10, w/v). After excess water removal via sieving, the crude protein content of the air-dried undigested samples was determined using the Kjeldahl method with duplicate measurements (AOAC, 2005). Equations for quantifying protein content in the undigested residue are shown in equations (6) and (7), respectively.

(6) $$\text{Nitrogen}\;\text{content}\;\left(\%\right)=\frac{\left({\text{V}}_{\text{s}}-{\text{V}}_{\text{b}}\right)\times M\times 14}{1000\times {\text{W}}_{\text{s}}}\times 100\%$$

where V_s_ represents the volume of HCl used for sample (mL), V_b_ stands for the volume of HCl used for blank (mL), M indicates the exact molarity of HCl (mol/L), and W_s_ denotes the actual weight of sample (g).

(7) Protein (%) = Nitrogen content (%) × Protein conversion factor

where the protein conversion factor used is 6.25.

#### 2.5.2 Profile of released amino acids after *in vitro* protein digestion

To explore the effects of hydrocolloid types and concentrations on the bioavailability of amino acids, digested supernatants were collected at the end (240 min) of the intestinal stage of *in vitro* digestion. The supernatants were filtered through a 0.22 μm hydrophilic non-pyrogenic polyether sulfone filter (Ministart® High Flow Filter Unit, Sartorius AG, Göttingen, Germany), and stored at −20 °C for subsequent analysis. Individual free amino acids in the filtered supernatant were analysed using AOAC 988.15 and 994.12 standard methods (AOAC, 2005). AOAC Tryptophan, due to its vulnerability to destruction by acid hydrolysis, was determined separately using high-performance liquid chromatography. All measurements were carried out in triplicates.

### 2.6 Confocal laser scanning microscopy

The microstructure of both PBFCs and commercial TFC were assessed using confocal laser scanning microscopy (CLSM) (A1R + si DUVB, Nikon, Tokyo, Japan) in fluorescent mode, employing the method derived from Grasso et al. (2022) with some adjustments. Slices of the samples, approximately 5 × 5 × 2 mm in size, were positioned on glass slides. Subsequently, 20 μL of Fast Green FCF (0.1 mg/mL in ethanol) and 20 μL of Nile Red (0.1 mg/mL in ethanol) were applied to each sample. The samples were then covered with a glass slip and incubated in darkness for 5 min. Observations were made at excitation wavelengths of 488 nm and 633 nm, respectively. These wavelengths caused the green and purple probes to fluoresce, and the resulting fluorescent images were superimposed to indicate the locations of protein and fat, respectively. All images were captured at 20 × of magnification.

### 2.7 Consumer sensory evaluation

The PBFC formulation that exhibited hardness and springiness statistically comparable to the commercial TFC was selected for a consumer sensory evaluation. A total of 125 staff and students from Singapore Institute of Technology (SIT), aged 18 and above, participated in the sensory session. Participants were guided to a private evaluation station in a designated sensory laboratory and directed to read an informational handout approved by SIT Institutional Review Board (Project number: 2022193) before the start of the evaluation. A preliminary questionnaire was issued to the panellists to screen for food allergies.

The samples were presented to the panellists in a balanced and randomised order, with both PBFC and TFC being coded and served during the same session. The serving temperature for both PBFC and TFC was maintained at 55 °C. Unsalted crackers and room-temperature water were provided to cleanse the palate. Panellists were instructed to assess the appropriateness and their degree of liking for attributes such as colour, hardness, springiness, oiliness, moistness, and overall liking. To express the appropriateness of these attributes, panellists employed a “Just About Right” (JAR) scale, which ranged from −2 to 2, with 0 indicating “Just About Right.” For indicating their liking, panellists used a five-point hedonic scale, which ranged from 1-"Dislike extremely” to 5-"Like extremely.” An example of the JAR and hedonic scale can be seen in Fig. S2.

### 2.8 Statistical analysis

The results of all the tests were presented as the mean and the standard error of the mean. The data were processed using Minitab statistical software, version 19.2020.2.0 (Minitab Inc., State College, PA, USA). To reveal statistically significant differences between multiple samples, one-way analysis of variance (ANOVA) was conducted, followed by Tukey's pairwise comparison module (*P* < 0.05) for normal data distribution. Welch's ANOVA was carried out for non-normal data distribution before performing Games-Howell pairwise comparison test (*P* < 0.05). For comparison of significant differences between two samples, two-sample *t*-test or paired *t*-test was conducted (*P* < 0.05). Before statistical analysis was done on the analytical test results, assumptions of ANOVA were checked, and outliers were removed from the data sets by employing Grubbs' test (*P* < 0.05) if necessary.

## 3 Results and discussion

### 3.1 Effect of hydrocolloid concentrations and types on HWHC and HOHC of protein-hydrocolloid powder blends

**Fig. 1:**
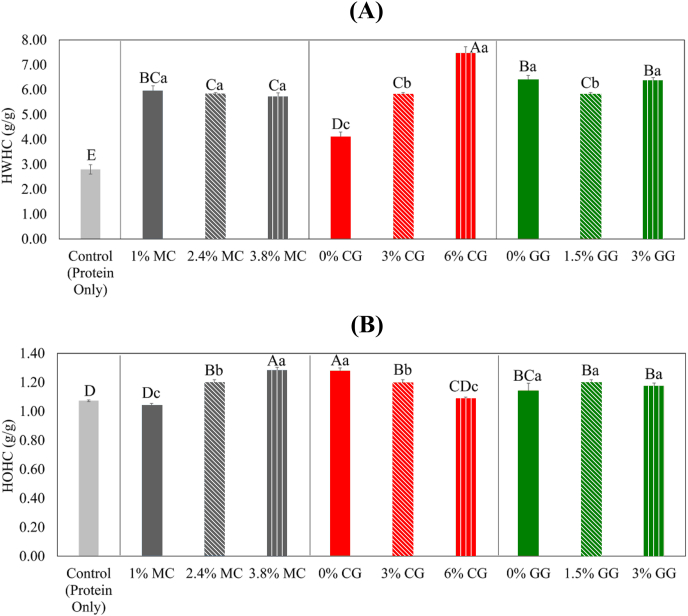
Heated water holding capacity (HWHC) (A) and heated oil holding capacity (HOHC) (B) of brown rice protein isolate and pea protein isolate (1:1) powder blended with different types and concentrations of hydrocolloids. Different capital letters indicate significant differences among the mean values across all samples. Different lowercase letters indicate significant differences among mean values for different concentrations within each hydrocolloid type. MC: methylcellulose; CG: curdlan gum; GG: high-acyl gellan gum. (For interpretation of the references to colour in this figure legend, the reader is referred to the Web version of this article.)

In Fig. 1A, it is evident that in the case of HWHC, all mixtures containing hydrocolloids displayed notably greater HWHC in comparison to the control group, which exclusively contained protein powder. Only CG exhibited an increase in HWHC with rising concentration. This phenomenon aligned with findings in previous studies where the addition of CG to plant-based nuggets, surimi, and processed meat products yielded similar results (Nishinari et al., 2021). The heightened water-holding capacity of CG can be attributed to its ability to form stronger molecular associations with denatured proteins via hydrogen bonds, resulting in a more compact and denser network (Nishinari et al., 2021). Additionally, there have been reports indicating that curdlan enhances the transfer of free water to bound water in surimi products (Wei et al., 2018).

As depicted in Fig. 1B, the HOHC of the control sample was markedly lower than that of all samples incorporating hydrocolloids, with the exceptions of 1% MC and 6% CG. An increase in MC concentration resulted in higher HOHC, while the opposite trend was observed for CG. Variances in GG concentration did not yield significant effects. The strong affinity of MC with oil can be attributed to the presence of hydrophobic methyl groups (Brady et al., 2017). Research has indicated that a lower degree of methyl group substitution leads to reduced oil absorption during deep frying (Primo-Martín et al., 2010). Furthermore, a prior study demonstrated that curdlan gum outperformed cellulose derivatives in terms of reducing oil uptake and mitigating moisture loss, thanks to its gel-forming properties induced by heat (Funami et al., 1999). These two properties, namely HWHC and HOHC, were of particular interest in our protein-hydrocolloid powder blends as they were deemed crucial for evaluating the mouthfeel of our final PBFC products.

### 3.2 Effect of hydrocolloid concentration and type on the physical properties of PBFC

#### 3.2.1 Textural properties

**Fig. 2:**
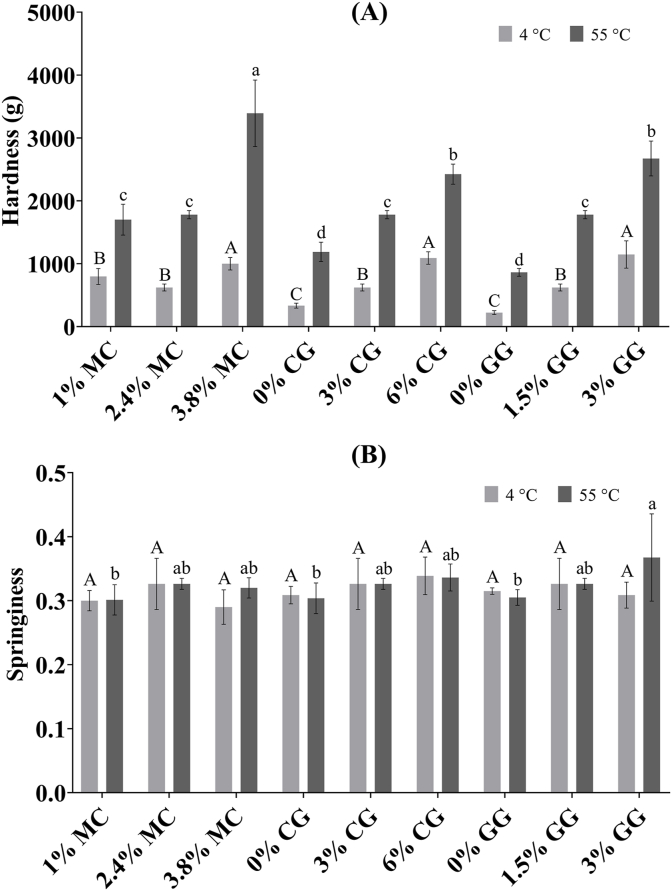
The hardness (A) and springiness (B) of plant-based fish cake analogues with different types and concentrations of hydrocolloids at both 4 °C and 55 °C. Different capital letters indicate significant differences among mean values across all samples at 4 °C. Different lowercase letters indicate significant differences among mean values across all samples at 55 °C. MC: methylcellulose; CG: curdlan gum; GG: high-acyl gellan gum.

Fig. 2A demonstrates a common pattern in which an increase in the concentration of hydrocolloids at both temperatures resulted in a significant rise in hardness. Generally, higher amounts of polysaccharides tend to promote stronger helix aggregation, thereby enhancing gel strength (Fittolani et al., 2020). Regarding MC variations, all samples demonstrated significantly higher hardness at 55 °C (1700 ± 244−3392 ± 529 g) compared to 4 °C (622 ± 55−999 ± 99 g). This can be attributed to the weakened hydrogen bonding between methoxy groups in MC and water molecules, alongside stronger hydrophobic interactions among polymer chains as the temperature increased (Ryu and McClements, 2024). A study by Jeong et al. (2023) similarly showed that the hardness of the canola oil emulsion gel did not vary significantly with MC ratios at 20 °C, but at 80 °C, gel hardness increased with higher MC concentration. This reversible heat-set gelling property was particularly evident at 3.8% MC, where the difference in hardness between 4 °C and 55 °C significantly widened.

In the presence of 2.4% MC (a concentration consistent across all CG and GG samples), both CG and GG variations led to an increase in hardness at 4 °C, reaching similar levels to that of TFC (1006 g at 4 °C, data not shown) in the 6% CG and 3% GG groups. Thus, it can be stated that although CG and GG couldn't completely counteract the reduction in hardness when transitioning from 55 °C to 4 °C due to MC, they did contribute to raising the hardness to a statistically similar level as TFC. The capability of CG and GG to complement the performance of MC at 4 °C can be attributed to the thermal-irreversible nature of CG gels when heated above 80 °C during fish cake processing and the cold-set characteristics of GG gels (Danalache et al., 2015; Jiang et al., 2020).

Referring to Fig. 2B, springiness did not exhibit a strong correlation with temperature or hydrocolloid concentration in most cases. It appeared that the thermal reversible property of MC had a less pronounced impact on springiness compared to its effect on hardness. The sample that displayed hardness and springiness statistically similar to TFC (TFC hardness: 1006 g at 4 °C, 1385 g at 55 °C; springiness: 0.32 at 4 °C, 0.33 at 55 °C, data not shown) at both temperatures was the one found in the 1% MC group (comprising 1% MC, 1.5% GG, and 3% CG). This particular sample was chosen for subsequent consumer sensory evaluation.

#### 3.2.2 Moisture increments of PBFC during fish cake making

**Fig. 3:**
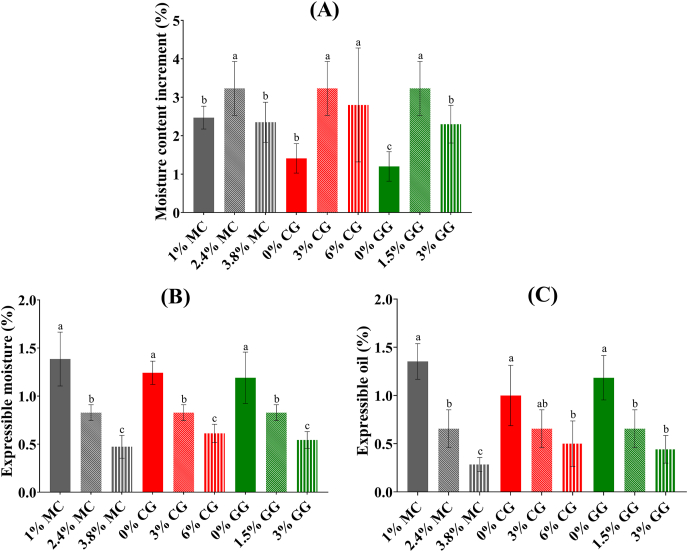
The moisture content increment (A), expressible moisture (B) and expressible oil (C) of plant-based fish cake analogues with different types and concentrations of hydrocolloids. Different letters indicate significant differences among mean values of different concentrations within each hydrocolloid type. MC: methylcellulose; CG: curdlan gum; GG: high-acyl gellan gum.

Fig. 3A shows that all PBFC samples gained moisture during processing regardless of hydrocolloid types and concentrations, as expected and evidenced by the HWHC results in Fig. 1A. A study from Funami et al. (1998) suggested that curdlan addition in meat promoted water absorption during heating processes. Another study reported an increase in moisture content of plant-based meat analogue by adding MC (Bakhsh et al., 2021). This is because hydrocolloids can form additional hydrogen bonds with water molecules due to the high amount of hydroxyl groups present in the polysaccharide. Additionally, hydrocolloids can help prevent moisture loss during the frying process. For example, when MC gels were heated, they created a protective layer that stopped moisture from escaping during frying (Bakhsh et al., 2021).

On the other hand, it is notable that the maximum water absorption was achieved when the concentration of hydrocolloids was within a mid-range, as indicated in Fig. 3A for all hydrocolloids. This observation of peak water absorption during the processing phase may be linked to the increased firmness of the processed product when higher concentrations of hydrocolloids were used, as supported by the results in Fig. 2A. This increased firmness could restrict the expansion of the product, impeding the permeation of water. Additionally, it has been observed that the water binding capacity could decrease when combinations of multiple polysaccharides created a state of excessive saturation (Wang et al., 2018).

#### 3.2.3 Expressible moisture and oil content

In Fig. 3B, it is evident that higher concentrations of hydrocolloids resulted in reduced expressible moisture. Generally, a lower expressible moisture content indicates enhanced gel strength due to a denser protein network or a greater number of water binding sites facilitated by the hydrocolloids (Jiao et al., 2019). Although previous research often linked expressible moisture to WHC (Wei et al., 2018), this study's HWHC results did not align with that interpretation. Therefore, it is believed that the expressible content was more significantly influenced by the textural properties as highlighted in Section 3.2.1.

As for expressible oil content, Fig. 3C shows a reduction in the expressible oil content, with a decrease from 1.35% in the 1% MC group to 0.28% in the 3.8% MC group, as the MC concentration increased. This phenomenon could be linked to MC's excellent HOHC attributed to its hydrophobic methyl groups (Brady et al., 2017). In the case of CG and GG, the same principle that explained the expressible moisture content could be applied to the current finding, which suggested that increased sample firmness led to reduced oil exudation, irrespective of the specific hydrocolloid types. In summary, all hydrocolloids demonstrated their ability to enhance water and oil retention in PBFC at higher concentrations.

#### 3.2.4 Rheological properties

**Fig. 4:**
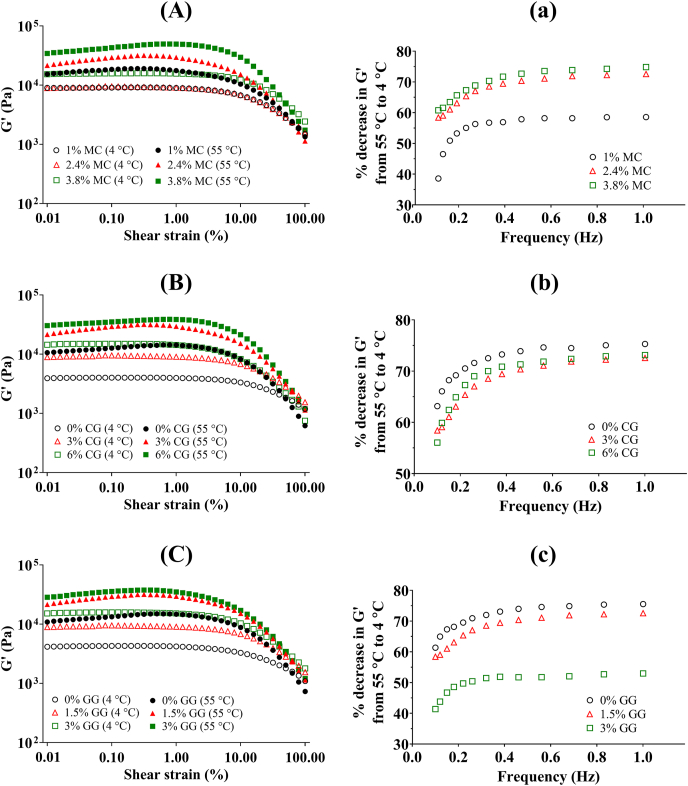
Shear strain dependence of storage modulus (G′) at 4 °C and 55 °C (A–C) and percentage decrease in G′ from 55 °C to 4 °C at various frequencies (a–c) for plant-based fish cake analogues with different types and concentrations of hydrocolloids. Data are presented as mean values. MC: methylcellulose; CG: curdlan gum; GG: high-acyl gellan gum.

In amplitude and frequency sweeps, significant attention was given to the storage modulus (G′) and loss modulus (G″) because they offer insights into the viscoelastic characteristics of the sample. In both tests, it was consistently observed that G′ values exceeded G″, indicating that the samples exhibited more elasticity or solid-like behaviour. Consequently, only the G′ values were displayed in Fig. 4.

##### 3.2.4.1 Amplitude sweep

In the amplitude sweep curves of Fig. 4A−C, a consistent trend emerged where an increase in the concentration of hydrocolloid led to higher stiffness (G′) of the gel. This indicates that the incorporation of hydrocolloids substantially reinforced the structural strength of the PBFC gel system. This finding aligned with our TPA results, which also demonstrated increased hardness at higher hydrocolloid concentrations as discussed in Section 3.2.1.

Furthermore, we observed that the gels exhibited greater strength at 55 °C compared to 4 °C, primarily due to the presence of thermally-reversible MC in all samples, in agreement with a prior study on the influence of MC addition in alginate-MC gel rheology (Eskens et al., 2020). A similar effect was also noted in soy protein gels induced by calcium sulfate, where higher concentrations of curdlan and gellan gum addition enhanced the gel's stiffness. This was attributed to curdlan gum's ability to form an elastic gel through hydrophobic interactions with soy protein under heating conditions, and gellan gum's physical-filling effect (Zhao et al., 2020).

##### 3.2.4.2 Frequency sweep

To investigate how temperature impacts both the strength and internal structure of PBFC gels prepared with varying concentrations of hydrocolloids, we analysed the results from frequency sweep tests. These results were presented to illustrate the percentage decrease in G′ values as the temperature was lowered from 55 °C to 4 °C. Frequency sweep tests were limited to a maximum frequency of 1 Hz, as some samples exhibited slippage at higher frequencies, introducing inaccuracies. Fig. 4a−c displays comparisons of the percentage decrease in G′ values from 55 °C to 4 °C at different concentrations for each hydrocolloid.

As shown in Fig. 4a, it is evident that higher concentrations of MC made the samples more prone to softening at lower temperatures. This finding under small oscillatory deformation corresponded well to the findings under large deformation during TPA as discussed in Section 3.2.1. The reason for this softening is the well-known thermo-reversible characteristic of MC gels, which tend to liquefy when cooled (Li, 2002). A similar pattern was observed in a batter formulated with MC, where the batter exhibited a fluid-like behaviour at 15 °C but transformed into a soft gel when heated to 60 °C (Sanz et al., 2005).

In contrast, Fig. 4b demonstrates that varying CG concentration did not significantly impact the decrease in G′ at cold temperatures. This was also supported by the TPA findings discussed in Section 3.2.1 and could be explained by the fact that curdlan gum exhibits thermo-irreversible nature when exposed to temperature above 80 °C (Jiang et al., 2020). In our study, such high temperature was reached during the PBFC manufacturing process. Consequently, the results for all three concentrations of CG were similar to that for the 2.4% MC concentration shown in Fig. 4a, which was the fixed concentration of MC present in all CG and GG samples, as the formation of the fish cake was not achievable without MC.

At higher GG concentrations, especially at 3%, there was a notable reduction in the percentage decrease of G′ when the temperature dropped, as seen in Fig. 4c. This finding suggested that incorporating GG may help in reducing textural changes during cooling. This corresponded well with the established knowledge that upon cooling, GG forms gels through an ordered double helical conformation, with weak interactions like van der Waals forces and hydrogen bonds facilitating helix associations. These interactions are reversible upon heating (Danalache et al., 2015). Although this property wasn't evident in the large deformation tests of TPA, incorporating GG tends to be an effective approach to supplement MC and boost gel strength in colder conditions. This approach may preserve the stability of PBFC and reduce the risk of quality deterioration due to temperature fluctuations.

### 3.3 In vitro protein digestion

#### 3.3.1 Protein digestion progress

**Fig. 5:**
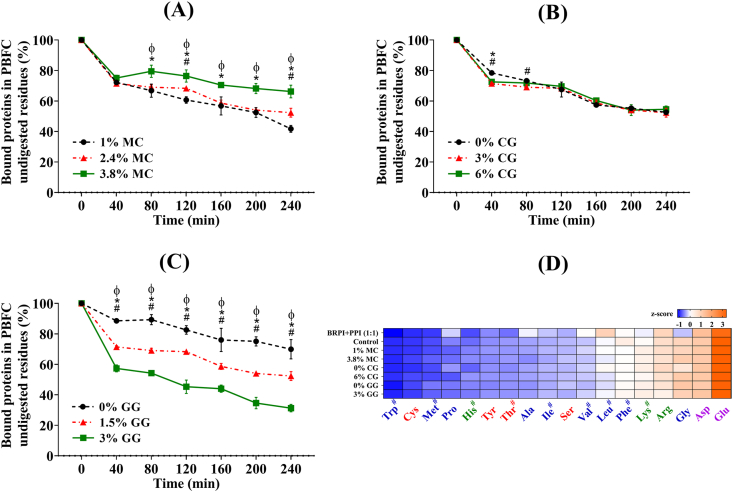
The protein digestion progress through indirect measurement of the percentage of bound proteins remaining in the undigested residues at different time points (A–C) and the heatmap (D) of the amino acids in the protein powder blend (BRPI + PPI 1:1) and plant-based fish cake analogues' digesta with different types and concentrations of hydrocolloids after *in vitro* digestion. Note: the marks #, * or φ indicate significant differences (*P* < 0.05) between: 1% MC and 2.4% MC, 1% MC and 3.8% MC, and 2.4% MC and 3.8% MC, respectively, at each time point (Subgraph A); 0% CG and 3% CG, 0% CG and 6% CG, and 3% CG and 6% CG, respectively, at each time point (Subgraph B); 0% GG and 1.5% GG, 0% GG and 3% GG, and 1.5% GG and 3% GG, respectively, at each time point (Subgraph C). Essential amino acids are marked with “#“, and purple, red, green and blue colour represents amino acids that are polar acidic, polar with no charge, polar basic and non-polar, respectively (Subgraph D). (For interpretation of the references to colour in this figure legend, the reader is referred to the Web version of this article.)

The extent of protein digestion of PBFC throughout the digestion process was examined by measuring the protein content of the undigested fish cake residues at different time intervals (Fig. 5). Fig. 5A illustrates a general trend of reduced protein digestion with higher concentrations of MC. This phenomenon could be explained by the increased hardness associated with higher amounts of MC, as demonstrated in Fig. 2A. This rationale was supported by a study conducted by Luo et al. (2021), which found that hard gels broke down more slowly than soft gels, making it more challenging for pepsin to hydrolyse the proteins. Alternatively, this reduced digestion may also result from the thickening of the gastrointestinal fluids caused by higher MC concentrations, as observed during the experiment. The thickening of digestive fluids can slow down the mixing of different components within the stomach (McClements, 2021). Moreover, since it has been reported that most enzyme active sites are hydrophobic, another possible mechanism in our study was that the hydrophobic methyl-rich regions in MC might bind to or interfere with the hydrophobic active sites of enzymes, thereby inhibiting the enzymes from hydrolysing proteins (Liu et al., 2020).

In contrast, as shown in Fig. 5B, the concentration of CG had little impact on the extent of protein digestion during most time points in the simulated gastric and small intestinal phases. Moreover, higher concentrations of GG even resulted in enhanced protein digestion (Fig. 5C). This phenomenon could be attributed to the neutral charge nature of CG. A study conducted by Borreani et al. (2016) revealed that the digestion rate of dairy proteins with a neutral polysaccharide showed minimal difference compared to the control group with no polysaccharide added, unlike the sample with negatively charged polysaccharide. Although MC also carries a neutral charge, this effect was not observed for MC, suggesting that gels formed by MC through hydrophobic interactions exerted a more significant influence than neutral charges in modulating protein digestion. On the other hand, a study by Kazemi-Taskooh and Varidi (2021) concerning whey protein-gellan hydrogel found that GG increased the porosity of the hydrogel, resulting in a faster release of iron during digestion, which aligned with our findings. Therefore, GG might have the ability to reduce aggregation among the protein particles or proteins bonded to other food components, creating a larger surface area accessible for enzymes, thus increasing the rate of digestion. To further confirm the trends we observed in protein digestion in relation to different hydrocolloid concentrations and types, we conducted a subsequent CLSM imaging test to gain morphological insights.

#### 3.3.2 Profile of amino acids released after *in vitro* digestion

To visualise the effect of hydrocolloid types and concentrations on the profiles of released amino acids, z-scores were used to plot the heatmap in Fig. 5D. Lower and higher relative concentrations (relative to the average concentration of all amino acids within each hydrocolloid concentration treatment group) were represented by blue and orange colour, respectively. We can see that across all PBFC groups, polar acidic amino acids exhibited relatively higher release after *in vitro* digestion compared to other amino acids, while tryptophan and cystine had the lowest release. However, hydrocolloid types and concentrations did not have a significant impact on the relative concentrations of the released amino acids, neither was there a discernible trend based on the polarity of the amino acids.

Given that all the protein in our PBFC came from BRPI and PPI, the amino acid profile of the unprocessed BRPI + PPI powder blend (5 g each) was also analysed as a reference. Notably, when other ingredients and processing steps were introduced to incorporate the protein powders into a fish cake product, the release pattern of certain amino acids in the fish cake system differed from that of the unprocessed protein powder blend alone. For example, there was a significantly higher proportion of glycine and lysine released among all the amino acids after digestion in PBFC compared to the proportions in the protein powders. In contrast, leucine, valine, alanine and proline had relatively lower release ratios in PBFC compared to their ratios in the protein blend. Since the released amino acid composition in PBFC did not completely align with that of the protein powders, it could be hypothesised that certain processes or intrinsic factors may have contributed to the observed differences.

### 3.4 Microstructural characteristics of PBFC

**Fig. 6:**
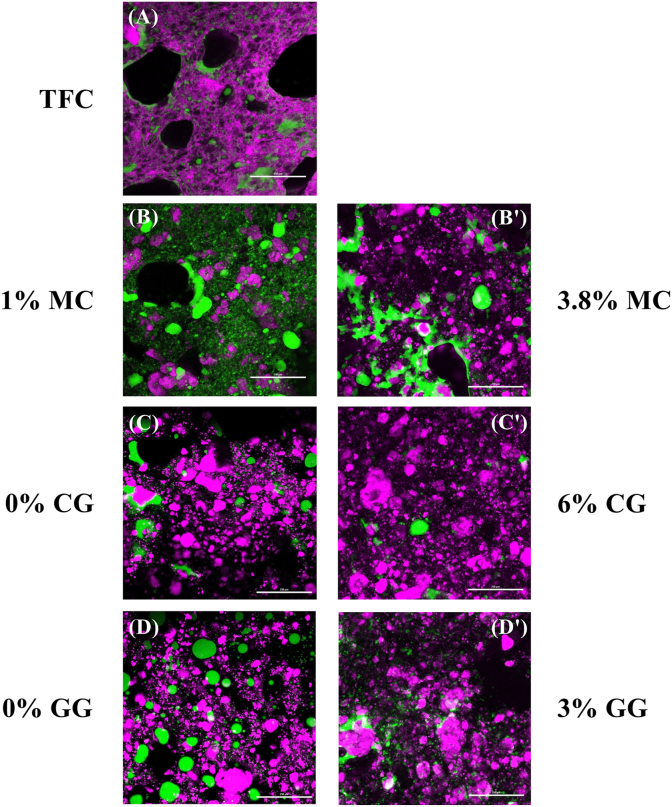
Confocal laser scanning microscopy images of commercial surimi-tofu fish cake (TFC) and plant-based fish cake analogues with different types and concentrations of hydrocolloids. MC: methylcellulose; CG: curdlan gum; GG: high-acyl gellan gum.

Microstructural variances in PBFCs containing MC, CG, and GG were observed using CLSM, as illustrated in Fig. 6. The green area represents the stained proteins, while the purple zone corresponds to the dyed oil droplets, which are the two primary components in our PBFC formulation.

The micrographs clearly revealed distinct characteristics in TFC where a continuous oil network surrounded the air bubbles (depicted as black holes) with trapped protein particles (Fig. 6A). In contrast, all PBFC samples exhibited scattered oil droplets, suggesting that the oil droplets resisted aggregation within the PBFC systems. This difference could lead to varying mouthfeel experiences between TFC and PBFC in the subsequent sensory evaluation.

Regarding protein structure, PBFC with a lower concentration of MC (1%) displayed a relatively uniform structure. In contrast, higher MC concentration (3.8%) resulted in some densely packed areas, indicating the presence of denser protein aggregates (Fig. 6BB’). This observation supported the occurrence of polymer associations between MC and amino acids of proteins through hydrophobic interactions, aligning with the textural and digestibility results.

For the CG and GG treatment groups, more homogeneous network structures were observed, with the oil droplets partially coalescing and reduced aggregation among protein particles when higher concentrations of CG (6%) and GG (3%) were applied during PBFC processing, compared to their respective control groups (Fig. 6CC’, DD’). Consequently, the protein digestibility of samples subjected to these two treatments remained uncompromised and even improved due to the increased surface areas of protein substrates.

### 3.5 Consumer sensory evaluation

**Fig. 7:**
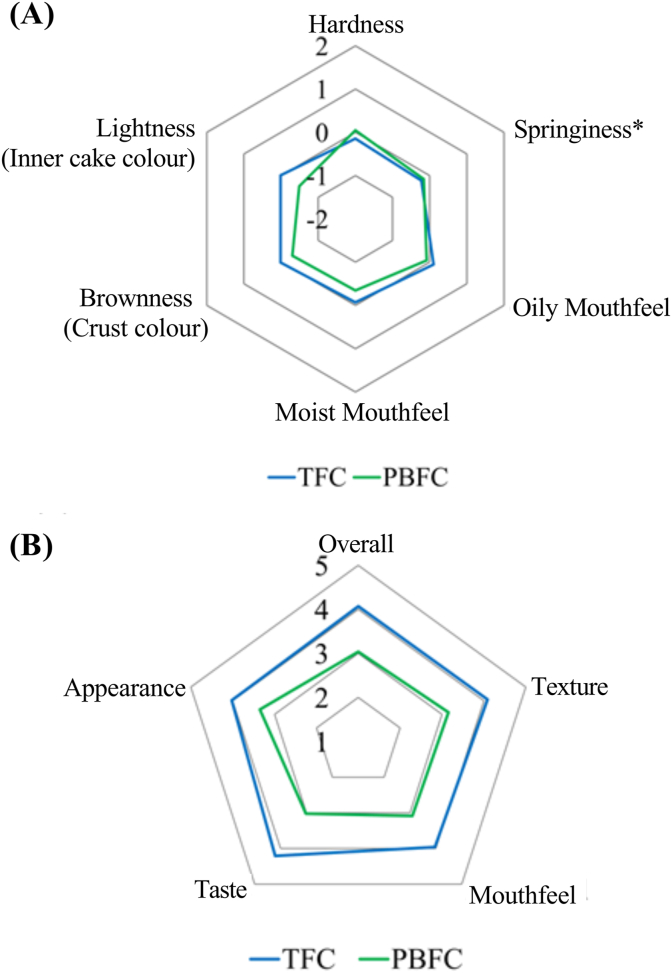
Consumer sensory evaluation of commercial surimi-tofu fish cake (TFC) and plant-based fish cake analogue (PBFC) formulated with 1% MC, 3% CG, and 1.5% GG. Data are presented as mean values. Result with no significant differences between TFC and PBFC is indicated with an asterisk (*).

According to the TPA results detailed in Section 3.2.1, PBFC formulated with 1% MC, 3% CG, and 1.5% GG exhibited hardness and springiness comparable to TFC. This specific formulation was chosen for consumer sensory evaluation. The sensory assessments were categorised based on attribute appropriateness using the JAR scale and overall liking using a 5-point hedonic scale, as illustrated in Fig. 7.

The appropriateness of attributes revealed that TFC closely matched JAR for all characteristics. However, PBFC only aligned with JAR in terms of hardness and springiness. Notably, only the springiness of PBFC was statistically similar to that of TFC. Although analytical TPA measurements indicated comparable hardness and springiness between PBFC and TFC, the sensory results indicated that consumers were more discerning, particularly in detecting differences in hardness. With a sample size of 125 participants, it can be concluded that the formulated PBFC demonstrated similar springiness appropriateness as the commercially available TFC.

In terms of liking, consumers consistently rated all attributes and the overall liking of PBFC one point lower than TFC, and this difference was statistically significant. This observation suggested that merely enhancing individual textural attributes was insufficient to enhance overall product liking. Another study exploring the relationship between structure, texture, and sensory perception of meat analogues corroborated this finding (Godschalk-Broers et al., 2022). The authors of that study proposed that to enhance liking, it was more effective and essential to manipulate sensory attributes with complex cross-modal interactions rather than solely improving individual textural characteristics.

## 4 Conclusion

Achieving appropriate physical properties like hardness, moisture content, and expressible moisture and oil content in PBFC necessitated a careful balance of hydrocolloid concentrations. The amount of expressed moisture and oil was more influenced by gel hardness than the inherent water and oil holding capacities of the hydrocolloids. Combining two or more hydrocolloids also proved effective in attaining the desired texture at both storage (4 °C) and serving (55 °C) temperatures, as demonstrated by the complementary gel properties of GG and MC indicated in the rheological results. Despite the positive impacts of hydrocolloids on physical properties, the types and concentrations of MC, CG and GG had varying effects on protein digestibility, and no consistent trends were observed between hydrocolloid types or concentrations and the release of amino acids after *in vitro* digestion. Although the underlying mechanisms of these findings are not fully validated, this study paves the way for further research on how interactions between proteins and polysaccharides, as well as their structural distribution in such systems, may influence physical properties and protein digestibility of PBFC. While further optimisation of the formulation is necessary, this study has also bridged the gap in consumer acceptance of plant-based products, as supported by the outcomes of the consumer sensory evaluation.

## Funding

This research was supported by the RIE2020/RIE2025 Singapore Food Story Theme 2 – 1st Alternative Protein Seed Challenge Grant (W20W2D0013) and Singapore Food Story R&D Programme Industry Alignment Fund Pre-positioning (IAF-PP) Theme 2 – Advanced Biotech-based Protein Production Grant (A21H7a0131 and H21H8a0005), administered by A*STAR.

## CRediT authorship contribution statement

**Felicia Zhi Wen Peh:** Conceptualization, Methodology, Investigation, Software, Visualization, Writing – original draft, Writing – review & editing. **Lin Zhao:** Methodology, Investigation, Software, Visualization, Writing – review & editing. **Yin Yin Chia:** Investigation, Writing – original draft. **Cheryl Kwoek Zhen Ng:** Methodology, Investigation. **Juan Du:** Funding acquisition, Conceptualization, Methodology, Project administration, Supervision, Writing – review & editing.

## Declaration of competing interest

The authors declare that they have no known competing financial interests or personal relationships that could have appeared to influence the work reported in this paper.

## Data Availability

Data will be made available on request.
