# Supplementary material for: Texture improvement and in vitro digestion modulation of plant-based fish cake analogue by incorporating hydrocolloid blends
Source: Curr Res Food Sci. 2024 May 23;8:100775. doi: 10.1016/j.crfs.2024.100775 (PMC11150973; doi:10.1016/j.crfs.2024.100775)
Supplement: Multimedia component 1 [file mmc1.docx]

**Table S1**

Chemicals and their respective concentrations used for the preparation of simulated salivary fluid (SSF), simulated gastric fluid (SGF) and simulated intestinal fluid (SIF).

| Chemicals | SSF (mmol/L)^a^ | SGF (mmol/L)^a^ | SIF (mmol/L)^a^ |
| --- | --- | --- | --- |
| KCl | 18.88 | 8.63 | 8.50 |
| NaHCO_3_ | 17.00 | 31.25 | 106.25 |
| KH_2_PO_4_ | 4.63 | 1.13 | 1.00 |
| MgCl_2_(H_2_O)_6_ | 0.19 | 0.12 | 0.41 |
| NaCl | - | 59.00 | 48.00 |
| (NH_4_)_2_CO_3_ | 0.08 | 0.63 | - |

^a^ Concentrations of stock solutions prepared were 1.25 times of the final concentration.

**Table S2**

Reagents and their amounts required for the simulated digestive fluids used at the oral, gastric and intestinal phase.

| Solutions | Oral Phase | Gastric Phase | Intestinal Phase |
| --- | --- | --- | --- |
| SSF (mL) | 2.40 | - | - |
| SGF (mL) | - | 4.80 | - |
| SIF (mL) | - | - | 9.60 |
| α-Amylase^a^ (mg) | 37.50 | - | - |
| Pepsin^b^ (mg) | - | 6.09 | - |
| Pancreatin^c^ (mg) | - | - | 24.82 |
| Amyloglucosidase (μL) | - | - | 48.00 |
| Bile^d^ (g) | - | - | 0.11 |
| Distilled Water (mL) | 0.59 | 1.06 | 2.30 |
| 0.3M CaCl_2_ (μL) | 15.00^#^ | 3.00^*^ | 24.00^^^ |
| 2M HCL^e^ (μL) | - | 141.00 | - |
| 2M NaOH^f^ (μL) | - | - | 30.00 |

^a^ Concentration of α-Amylase in SSF achieved is 15.625 mg/mL to achieve final enzyme activity of 75 U/mL.

^b^ Concentration of pepsin in SGF achieved is 1.269 mg/mL to achieve final enzyme activity of 2000 U/mL.

^c^ Concentration of pancreatin in SIF achieved is 2.585 mg/mL to achieve final enzyme activity of 100 U/mL based on trypsin activity.

^d^ Concentration of bile required in intestinal phase is 10.00 mM.

^e^ Pre-tested volume of HCL required to achieve PH 3.0 in gastric phase.

^f^ Pre-tested volume of NaOH required to achieve PH 7.0 in intestinal phase.

^#^ Final concentration of 0.3M CaCl_2_ required in oral phase is 1.50 mM.

^*^ Final concentration of 0.3M CaCl_2_ required in gastric phase is 0.15 mM.

^^^ Final concentration of 0.3M CaCl_2_ required in intestinal phase is 0.605 mM.


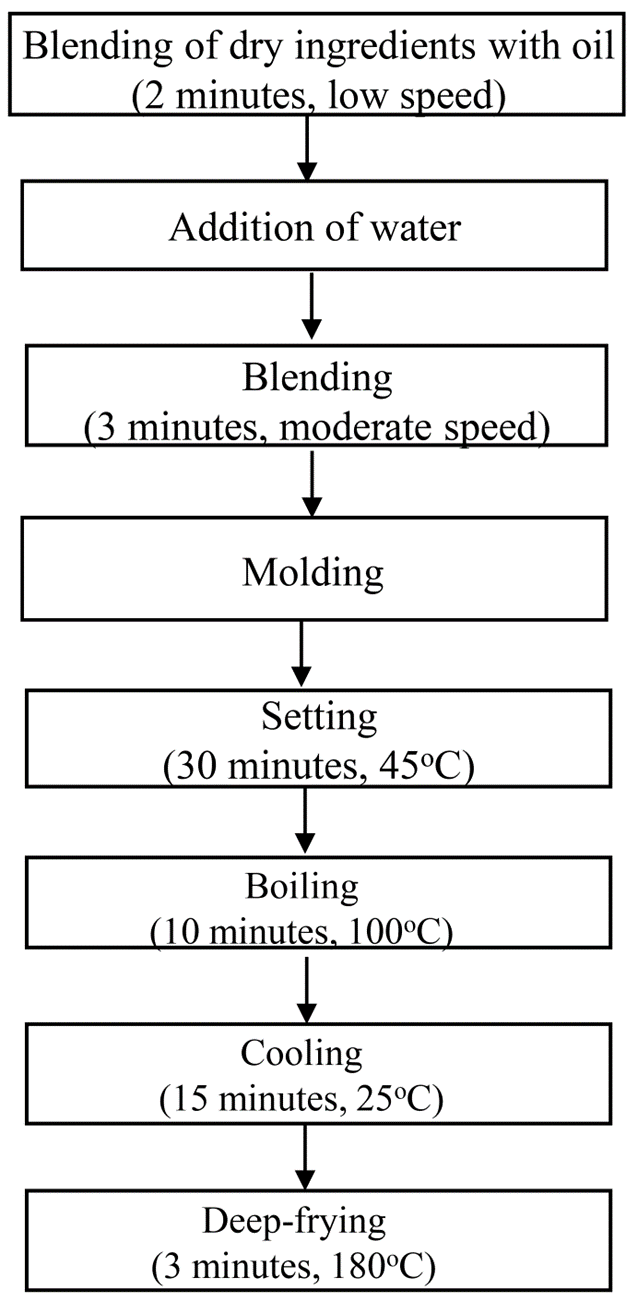


**Fig. S1.** Process flow diagram of making PBFC.


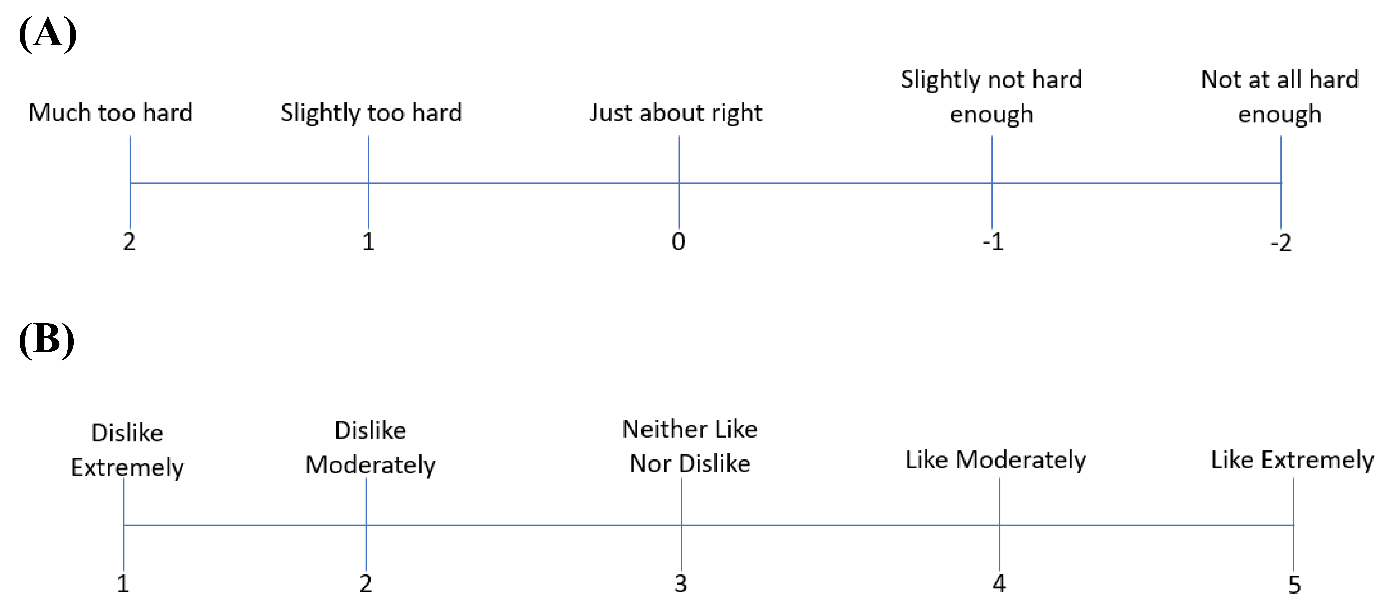


**Fig. S2.** An example of Just-About-Right scale (A) and 5-point hedonic scale (B) used in the consumer sensory evaluation questionnaire.
